# Supplementary material for: Evaluation of Drug Interactions in Patients Treated with DAAs for Hepatitis C Therapy with Comorbidities and Cardiovascular Issues—A Delphi Consensus Project
Source: J Clin Med. 2022 Nov 25;11(23):6946. doi: 10.3390/jcm11236946 (PMC9737105; doi:10.3390/jcm11236946)
Supplement: Supplementary file 1 [file jcm-11-06946-s001.zip › Supplementary Materials S1-Questionnaires.pdf]

Supplementary Materials S1 - Questionnaires

QUESTIONNAIRE 1

SECTION Respondent Profile

a. First Name/Family name: ....
b. Year of Birth: ....
c. Year of Graduation: ....
d. Specialization: ....
e. Facility where you work: ....
   The facility where you work is [you can select multiple answers]
   1. ☐ University
   2. ☐ Public hospital
   3. ☐ Private hospital
   4. ☐ Territorial service (outpatient clinic)
f. Prevailing Scope of Practice:
g. How many years you have been involved in your prevailing scope of practice: ....
h. Your role in the facility: ....
i. Are you a member of one or more scientific societies?
   ❍ Yes ❍ No

If yes, which ones? ....

SECTION Delphi Questionnaire

1. What tool do you use to assess the risk of drug interactions before starting HCV therapy?
[express the level of implementation for each answer choice by selecting a score from 1 (lowest grade) to 9 (highest grade)]

a.	EASL 2020 Recommendations	1 2 3 4 5 6 7 8 9
b.	University of Liverpool database	1 2 3 4 5 6 7 8 9
c.	Package leaflet	1 2 3 4 5 6 7 8 9
d.	Personal experience	1 2 3 4 5 6 7 8 9
e.	Medscape	1 2 3 4 5 6 7 8 9

2. What are the most frequent comorbidities among your patients with hepatitis C?
[you can select multiple answers]

a. ☐ Anxiety
b. ☐ COPD
c. ☐ Ischemic heart disease
d. ☐ Depression
e. ☐ Diabetes
f. ☐ Dyslipidemia
g. ☐ Epilepsy
h. ☐ Atrial fibrillation
i. ☐ Renal failure
j. ☐ Hypertension
k. ☐ Thyroid disease
l. ☐ Parkinson's disease

3. What are the most frequent co-medications among your patients?
[you can select multiple answers]

a. ☐ Analgesics/NSAIDs
b. ☐ Anxiolytics / Antidepressants
c. ☐ Antacids
h. ☐ Chemotherapeutic agents
i. ☐ Proton pump inhibitors
j. ☐ DOAC

d. ☐ Antiplatelet
e. ☐ Antiarrhythmics
f. ☐ Antiepileptics
g. ☐ Antihypertensives
k. ☐ Self-medication products
l. ☐ Statins
m. ☐ Warfarin

4. **HCV infection is a risk factor for the development of cardiovascular disease.**
[please indicate your level of agreement with this statement by selecting a score from 1 (complete disagreement) to 9 (maximum agreement)]
1 2 3 4 5 6 7 8 9

5. **Eliminating viral infection with direct antiviral drugs reduces the risk of cardiovascular disease development or progression.**
[please indicate your level of agreement with this statement by selecting a score from 1 (complete disagreement) to 9 (maximum agreement)]
1 2 3 4 5 6 7 8 9

6. **Which sequence do you consider a priority for a patient with HCV and cardiovascular disease?**
[please indicate the order of importance you attach to the proposed elements by assigning value 1 to the highest priority sequence and value 4 to the lowest priority sequence]

a. Consider the CV effects of HCV treatment	1	2	3	4
b. Framing the CV risk profile	1	2	3	4
c. Considering pharmacological treatment of CVD compatible and with fewer drug interactions with HCV therapy	1	2	3	4
d. Shared management between cardiologist and hepatologist / infectologist	1	2	3	4

7. **In the case of concomitant HCV infection and CVD, discontinuation of antihypertensives, even for 8-12 weeks, may lead to an increase in blood pressure variability and, thus, an increased risk of developing cardiovascular complications.**
[please indicate your level of agreement with this statement by selecting a score from 1 (complete disagreement) to 9 (maximum agreement)]
1 2 3 4 5 6 7 8 9

8. **In the case of concomitant HCV infection and CVD, replacing antihypertensives, even for 8-12 weeks, with a drug that is not equally potent may lead to an increase in blood pressure variability and thus an increased risk of developing cardiovascular complications.**
[please indicate your level of agreement with this statement by selecting a score from 1 (complete disagreement) to 9 (maximum agreement)]
1 2 3 4 5 6 7 8 9

9. **In the case of concomitant HCV infection and CVD, changing the dosage of the current antihypertensive drug may lead to an increase in blood pressure variability and thus an increased risk of developing cardiovascular complications.**
[please indicate your level of agreement with this statement by selecting a score from 1 (complete disagreement) to 9 (maximum agreement)]
1 2 3 4 5 6 7 8 9

10. **In cases of concomitant HCV infection and CVD, discontinuation of statins, even for 8-12 weeks, may lead to an increased risk of developing cardiovascular complications.**
[please indicate your level of agreement with this statement by selecting a score from 1 (complete disagreement) to 9 (maximum agreement)]

1 2 3 4 5 6 7 8 9

11. **In the case of concomitant HCV infection and CVD, replacing statins, even for 8-12 weeks, with a drug that is not equally potent may lead to an increased risk of developing cardiovascular complications.**
[please indicate your level of agreement with this statement by selecting a score from 1 (complete disagreement) to 9 (maximum agreement)]
1 2 3 4 5 6 7 8 9

12. **In the case of concomitant HCV infection and CVD, changing the dosage of ongoing statins may lead to an increased risk of developing cardiovascular complications.**
[please indicate your level of agreement with this statement by selecting a score from 1 (complete disagreement) to 9 (maximum agreement)]
1 2 3 4 5 6 7 8 9

13. **In cases of concomitant HCV infection and CVD, discontinuation of DOACs, even for 8-12 weeks, may lead to an increased risk of developing cardiovascular complications.**
[please indicate your level of agreement with this statement by selecting a score from 1 (complete disagreement) to 9 (maximum agreement)]
1 2 3 4 5 6 7 8 9

14. **In the case of concomitant HCV infection and CVD, replacing DOACs, even for 8-12 weeks, with a drug that is not equally potent may lead to an increased risk of developing cardiovascular complications.**
[please indicate your level of agreement with this statement by selecting a score from 1 (complete disagreement) to 9 (maximum agreement)]
1 2 3 4 5 6 7 8 9

15. **In the case of concomitant HCV infection and CVD, changing the dosage of ongoing DOACs may lead to an increased risk of developing cardiovascular complications.**
[please indicate your level of agreement with this statement by selecting a score from 1 (complete disagreement) to 9 (maximum agreement)]
1 2 3 4 5 6 7 8 9

16. **For the management of hypertension in the HCV patient, ACE inhibitors should only be administered if necessary and preferably in combination with sofosbuvir / velpatasvir (SOF/VEL) and not in combination with glecaprevir / pibrentasvir (GLE/PIB).**
[please indicate your level of agreement with this statement by selecting a score from 1 (complete disagreement) to 9 (maximum agreement)]
1 2 3 4 5 6 7 8 9

17. **In the case of combined therapy with antihypertensives, SOF/VEL is the most effective and safe choice.**
[please indicate your level of agreement with this statement by selecting a score from 1 (complete disagreement) to 9 (maximum agreement)]
1 2 3 4 5 6 7 8 9

18. **In HCV patients, direct anticoagulants (DOAC), which have a simplified mechanism and an excellent efficacy/safety ratio, are preferred.**
    a. Yes

b. No
c. Only in the presence of severe liver failure

19. In the case of polypharmacotherapy, it is important not to change the full doses of the current drug, favoring the choice of the antiviral with the least interference.
[please indicate your level of agreement with this statement by selecting a score from 1 (complete disagreement) to 9 (maximum agreement)]
1 2 3 4 5 6 7 8 9

20. Cardiovascular risk assessment through risk maps must take into account the latest evidence, considering additional elements such as modifiers and comorbidities.
[please indicate your level of agreement with this statement by selecting a score from 1 (complete disagreement) to 9 (maximum agreement)]
1 2 3 4 5 6 7 8 9

21. The drug interaction profile between HCV antiviral drugs and the main statins used is similar.
[please indicate your level of agreement with this statement by selecting a score from 1 (complete disagreement) to 9 (maximum agreement)]
1 2 3 4 5 6 7 8 9

22. Stopping the use of a statin for 8-12 weeks does not lead to a significant increase in CVD risk in primary prevention.
[please indicate your level of agreement with this statement by selecting a score from 1 (complete disagreement) to 9 (maximum agreement)]
1 2 3 4 5 6 7 8 9

23. Discontinuation of a statin for 8-12 weeks can also be tolerated in secondary prevention.
[please indicate your level of agreement with this statement by selecting a score from 1 (complete disagreement) to 9 (maximum agreement)]
1 2 3 4 5 6 7 8 9

24. The management of drug interactions with HCV antiviral drugs is straightforward and does not require special multidisciplinary approaches.
[please indicate your level of agreement with this statement by selecting a score from 1 (complete disagreement) to 9 (maximum agreement)]
1 2 3 4 5 6 7 8 9

QUESTIONNAIRE 2

A. In cases of concomitant HCV infection and ischemic heart disease, discontinuation of antithrombotic therapy, even for 8-12 weeks, may lead to an increased risk of developing cardiovascular complications.
[please indicate your level of agreement with this statement by selecting a score from 1 (complete disagreement) to 9 (maximum agreement)]
1 2 3 4 5 6 7 8 9

B. **In the case of concomitant HCV infection and ischemic heart disease, de-escalation of antithrombotic therapy, even for 8-12 weeks, with a drug that is not equally potent may lead to an increased risk of developing cardiovascular complications.**
[please indicate your level of agreement with this statement by selecting a score from 1 (complete disagreement) to 9 (maximum agreement)]
1 2 3 4 5 6 7 8 9

C. **In cases of concomitant HCV infection and ischaemic cardiovascular disease, reducing the dosage of ongoing antithrombotic therapy may lead to an increased risk of developing cardiovascular complications.**
[please indicate your level of agreement with this statement by selecting a score from 1 (complete disagreement) to 9 (maximum agreement)]
1 2 3 4 5 6 7 8 9

D. **For the management of high blood pressure in HCV patients, ACE inhibitors should be administered in combination with sofosbuvir / velpatasvir (SOF/VEL).**
[please indicate your level of agreement with this statement by selecting a score from 1 (complete disagreement) to 9 (maximum agreement)]
1 2 3 4 5 6 7 8 9

E. **For the management of hypertension in HCV patients, ACE inhibitors should be administered in combination with glecaprevir / pibrentasvir (GLE/PIB).**
[please indicate your level of agreement with this statement by selecting a score from 1 (complete disagreement) to 9 (maximum agreement)]
1 2 3 4 5 6 7 8 9

F. **Temporary suspension of a cardiovascular therapy that the patient is taking for a chronic condition for the period of antiviral treatment entails the risk that the patient will prolong the suspension beyond the period indicated.**
[please indicate your level of agreement with this statement by selecting a score from 1 (complete disagreement) to 9 (maximum agreement)]
1 2 3 4 5 6 7 8 9

G. **Temporary substitution of a cardiovascular therapy that the patient is taking for a chronic condition for the period of antiviral treatment entails the risk that the patient will extend the interruption beyond the period indicated.**
[please indicate your level of agreement with this statement by selecting a score from 1 (complete disagreement) to 9 (maximum agreement)]
1 2 3 4 5 6 7 8 9

H. **Temporarily altering the dosage of a cardiovascular therapy a patient is taking for a chronic condition for the period of antiviral treatment carries the risk that the patient will extend the interruption beyond the period indicated.**
[please indicate your level of agreement with this statement by selecting a score from 1 (complete disagreement) to 9 (maximum agreement)]
1 2 3 4 5 6 7 8 9
