# Supplementary material for: Evaluation of Drug Interactions in Patients Treated with DAAs for Hepatitis C Therapy with Comorbidities and Cardiovascular Issues—A Delphi Consensus Project
Source: J Clin Med. 2022 Nov 25;11(23):6946. doi: 10.3390/jcm11236946 (PMC9737105; doi:10.3390/jcm11236946)
Supplement: Supplementary file 1 [file jcm-11-06946-s001.zip › Supplementary Materials S2-Contributors.pdf]

Supplementary Materials S2 - List of contributors

Ester Marina Cela, UOC Gastroenterologia Ospedaliera, Ospedali Riuniti di Foggia; Paolo Del Poggio, UO Gastroenterologia ed Endoscopia, Policlinico San Marco, Zingonia (BG); Claudio Ferri, Università degli Studi dell'Aquila; Mimmo Gabrielli, Casa di Cura Pio XI, Roma; Adriano Pellicelli, San Camillo Forlanini, Roma; Marcello Persico, UOC Clinica Medica ed Epatologica, AOU San Giovanni di Dio e Ruggi d'Aragona, Salerno; Emanuele Pontali, SC Malattie Infettive, Ospedali Galliera, Genova; Dante Romagnoli, Unità di Gastroenterologia, Policlinico di Modena, Modena; Mauro Viganò, UO Epatologia, Ospedale S. Giuseppe, Milano.
